# Fabrication and characterization of sustainable composites from animal fibers reinforced unsaturated polyester resin

**DOI:** 10.1016/j.heliyon.2024.e33441

**Published:** 2024-06-23

**Authors:** Md. Farhad Ali, Md. Sahadat Hossain, Israt Jahan Lithi, Samina Ahmed, A.M. Sarwaruddin Chowdhury

**Affiliations:** a Institute of Leather Engineering and Technology, University of Dhaka, Dhaka, 1000, Bangladesh; b Glass Research Division, Institute of Glass & Ceramic Research and Testing, Bangladesh Council of Scientific and Industrial Research (BCSIR), Dhaka, 1205, Bangladesh; c Department of Applied Chemistry and Chemical Engineering, Faculty of Engineering and Technology, University of Dhaka, Dhaka, 1000, Bangladesh

## Abstract

The research involves developing eco-friendly polymer composites by combining synthetic unsaturated polyester resin (UPR) with treated and untreated leather fibers (LF), cow hair fibers (CHF), and chicken feather fibers (CFF). By using these natural fibers instead of synthetic polymer, we aim to reduce the environmental impact while finding new purposes for waste materials from the poultry and tannery industries which would otherwise end up in garbage. The fibers were incorporated into the resin matrix at various weight percentages such as 2, 5, 7, 10, 12, and 15 % (w/w). Additionally, the properties of composites were improved by the addition of different types of inorganic nanoparticles, like CaCO_3,_ Al_2_O_3_, and ZnO to the UPR matrix. These composites where inorganic materials were added as filler revealed better results than the neat composites. The composites showed the maximum overall mechanical properties in the bending modulus (BM), bending strength (BS), tensile modulus (TM), and tensile strength (TS) when 5 % of cow hair and chicken feather mixed fiber was used and ZnO was added as filler compared to the other composites. The highest values 4400.415, 68.91, 1788.74, and 34.95 N/mm^2^ of BM, BS, TM, and TS respectively, were found for the CFF + CHF + UPR + ZnO composite. Scanning electron microscopy (SEM) and Fourier transform infrared spectroscopy (FTIR) supported mechanical rather than chemical connections between fiber and UPR. Ionizing gamma radiation-modified fiber exhibited superior tensile characteristics when 5 % of cow hair and chicken feather mixed fiber was used.

## Highlights

• Sustainable composites were fabricated using waste animal fibers.
• Mechanical properties were explored.
• Mechanical properties were increased using gamma radiation.
• Surface morphology and functional groups were investigated.

## 1 Introduction

Natural fibers derived from animals, minerals, or plants are distinct from synthetic fibers due to their cost-effectiveness, low density, and biodegradability [1]. Various research studies have highlighted the exceptional physical and mechanical properties of feathers. They have demonstrated remarkable resilience against mechanical stress and have proven their ability to withstand a variety of thermal and chemical treatments without undergoing lasting damage. This is only possible because of the keratin present in feathers, which provides strength and toughness and helps to protect the organism from mechanical damage and environmental stress. Keratin structures present in feathers in birds and fur in mammals are also shown to help maintain a stable internal temperature in varying environmental conditions.

Unsaturated polyester resins are made through a cross-linking reaction between vinyl-type monomers and liquid unsaturated polyester, resulting in the formation of polymers [2]. These are used in many applications, like the production of automotive parts, construction, and composite material, in the marine industry due to their resistance to water, chemicals, and weathering. Unsaturated polyester resin forms composite materials with excellent strength-to-weight ratios when combined with reinforcing fibers such as fiberglass or carbon fiber. Due to fibrous solid protein keratin, chicken feathers or cow hairs can be used to produce composite materials mixed with unsaturated polyester resin. Keratin is shown to have strong mechanical properties [3].

Every year, chicken processing plants dump billions of kilos of feathers, which is a sizeable quantity of solid waste. Feathers account for a sizable portion of the garbage produced by the chicken business. The maximum weight of a bird's feather is 125 g; therefore, around 3000 tons of feather waste are created worldwide each week [4]. Thus, Innovative methods have been created for underdeveloped countries to use animal fibers as raw materials to generate high-value products at competitive prices. Waste leather fibers are currently used to manufacture products that impact the environment, such as gelatin and glue, but chicken feathers and cattle hair are still not used in our country. Animal fiber-reinforced composites possess great promise for expanded usage and have a broad array of potential applications in different sectors such as construction, automotive, textiles and consumer products. Composite materials based on animal fiber can be used to replace various plastic items made of nondegradable polymer. The stability and expansion of our economy can benefit significantly from the use of composites made from animal fibers and also promote eco-friendly manufacturing practices. Molding, furniture, windows, doors, bathtubs, partition boards, storage tanks, toys, etc., may all benefit from its usage. This research aims to produce composite materials from chicken feathers and cow hair fibers, improving environmental sustainability, reducing carbon footprint, and reducing waste production.

## 2 Materials

The raw materials utilized in this research project included Waste Leather Fiber (LF), Cow Hair Fiber (CHF), and Chicken Feather Fiber (CFF). The chicken feather fibers were obtained from a local poultry farm situated nearby, while the black cow hair fiber and shavings were collected from the leather industry in Hemayetpur, Savar, Dhaka, Bangladesh.

## 3 Chemicals

The following materials were obtained for the project: ZnO, Al_2_O_3_, CaCO_3_, and KOH from Merck in Germany, as well as Hardener (MEKP) and unsaturated polyester resin (URP) from Pidilite Industries Ltd. in India. These were purchased from Lucky Acrylic Ltd. in Hatkhola Chemical Market, Dhaka. All the chemicals were commercial grade.

## 4 Methods

The leather and tannery industries supplied the fibers used to create unsaturated polyester resin composites with cow hair. The composites were made using the hand lay-up method. We tested different amounts of cow hair in the composites- 2, 5, 7, 10, 12, 15, and 20 % of the weight. The mechanical properties like bending modulus, bending strength, tensile modulus and tensile strength were measured. Additionally, SEM, water uptake analyses and FTIR were conducted. The finest results were obtained for composite with 5 % cow hair [5]. The commercial application of animal fiber (human hair) waste is observed in India. The composites were made by using human hair with polypropylene (PP). Two roll mills combined human hair and polypropylene at weights of 5 %, 10 %, and 15 %. When compression molding, certain conditions had to be met. The hair-reinforced polypropylene (PP) composites showed better Impact Strength and Flexural Strength than the control sample polypropylene [6]. The polymer composites were prepared with the collected cow hair fibers from the tannery wastes and leather industries and Low-Density Polyethylene (LDPE). The LDPE was recycled from water sachets and other plastic wraps made of LDPE. Melt combined with scraps and using a hot compression molding process yielded composites. Cow hair fibers were treated with 0.2 M KOH, 0.2 M NaOH, and 0.2 M H_2_O_2_ [7] to improve their adhesive qualities. To observe the mechanical and physical properties, the fibers' loading was varied from 0 to 50 % of the weight with intervals of 10 % weight. Chemically treated fibers added composites showed better results [8].

The “raw” chicken feathers were cleaned carefully with detergent and dried naturally under the sun. After the cleaning thoroughly, machines were used to separate them from the quills. The feathers were then divided into two piles: one containing useable fiber for future textile production and the other containing large feather and quill scraps [9]. To create nonwoven batting, the fibers from chicken feathers were combined with binder fibers (sheath/core construction). The composites were prepared using polyester resin and reinforced with cow hair from Zebu breed cattle in Nigeria. The hairs were collected from the tails portion of the cattle and were about 10 cm long. They treated fibers with NaOH solution. The composites were made using the hand lay-up technique at ambient temperature. The fiber loading was 0 %, 5 %, 10 %, 15 %, and 20 % of the total weight. For the matrix's random dispersion, short fibers were required. NaOH was used to treat some cow hair fibers, which were then chopped to 10 mm in length. The composites were created using an open-mold manufacturing approach. The samples underwent two curing processes: the first after manufacture and the second 27 days before testing. Cow hair fibers were treated with 0.2 M KOH, 0.2 M NaOH and 0.2 M H_2_O_2_ to improve their adhesive qualities. To observe the mechanical and physical properties, the fibers' loading was varied from 0 to 50 % of the weight with intervals of 10 % weight.

### 4.1 Composite fabrication

**Fig. 1:**
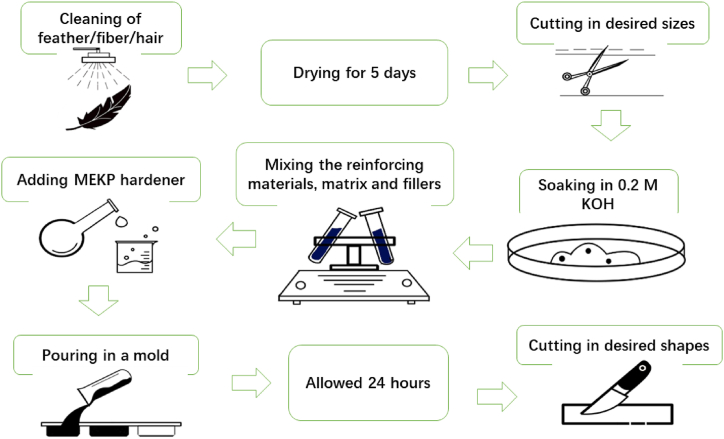
Composite fabrication process.

Chicken feathers (CFF), cow hairs (CHF), and discarded leather (LF) all underwent many steps outlined in Fig. 1 for the purifying process. The materials were first washed with distilled water and detergent to remove any dirt and bacteria. Subsequently, they were then dried in sunlight for 12 h and placed in an oven at 30^o^C for 5 days to remove surface contaminants. This thorough process ensured that no impurities and pollutants were present, and thus, they were ready for analysis.

A cutting instrument from FRITSCH Industries tr. 8, 55743 Idar-Oberstein, Germany, was used to cut the materials into 5 mm segments, and a fraction of the samples underwent a 2-h soak in 0.20 M KOH at 50 ^o^C and then dried at 60 ^o^C for 5 h. The remaining portion was left unattended.

A glass slab was placed on a table, on top of which a sheet of transparent plastic paper (Mica) was positioned. In a sterile plastic beaker, a mixture comprising MEKP hardener, unsaturated polyester resin (URP), treated and untreated chicken feather fibers, and chemical components (ZnO, Al_2_O_3_, and CaCO_3_) was thoroughly mixed using a glass rod. The mixture was then poured onto a plastic paper. After ensuring the removal of trapped air bubbles, the mixture was covered with Mica and a glass rod was used to uniformly distribute the material, which was allowed to solidify for 24 h. Then, they were transferred to polythene bags for storage.

Using the hand lay-up technique, a range of composite materials denoted as CFF-UPR, CHF-UPR, and LF-UPR were made by integrating different proportions of fiber components (2 %, 5 %, 7 %, 10 %, 12 %, and 15 % by weight) with unsaturated polyester resin (UPR) matrix as shown in Table 1. This process involved carefully layering and integrating the chosen fiber percentages with the UPR matrix to form reinforced composites. Ionizing gamma radiation was applied to a section of the produced composites to enhance their physio-mechanical characteristics [10].

**Table 1:** Compositions of the composites.

Composite	Chicken Feather Fiber/Cow Hair fiber/leather fiber	UPR	ZnO/Al_2_O_3_/CaCO_3_	Composite	Treated Chicken Feather Fiber/Treated Cow Hair fiber/Treated leather fiber	UPR	ZnO/Al_2_O_3_/CaCO_3_
1	2 %	98 %		2	2 %	98 %	
3	5 %	95 %		4	5 %	95 %	
5	7 %	93 %		6	7 %	93 %	
7	10 %	90 %		8	10 %	90 %	
9	12 %	88 %		10	12 %	88 %	
11	15 %	85 %		12	15 %	85 %	
	When oxide and/or carbonates were added						
1	2 %	97 %	1 %	2	2 %	97 %	1 %
3	5 %	94 %	1 %	4	5 %	94 %	1 %
5	7 %	92 %	1 %	6	7 %	92 %	1 %
7	10 %	89 %	1 %	8	10 %	89 %	1 %
9	12 %	87 %	1 %	10	12 %	87 %	1 %
11	15 %	84 %	1 %	12	15 %	84 %	1 %

## 5 Results and discussion

Mechanical Properties Test of Polymer Composites.

### 5.1 Tensile strength analysis

**Fig. 2:**
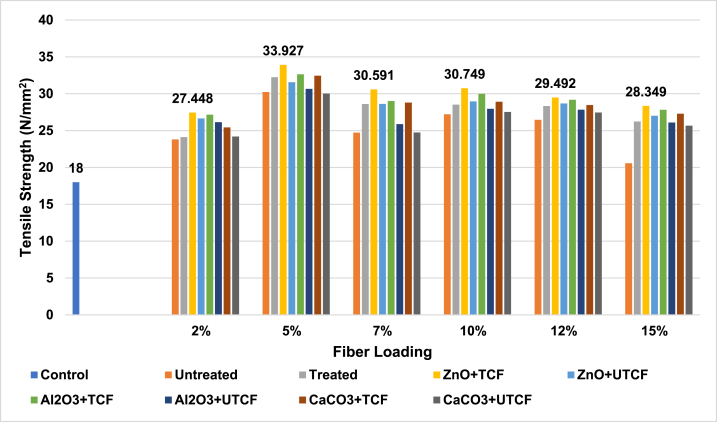
Tensile Strength peaks of Control Sample, UTCFF + UPR, TCFF + UPR, TCFF + UPR + ZnO, TCFF + UPR + Al_2_O_3_ TCFF + UPR + CaCO_3_, UTCFF + UPR + ZnO, UTCFF + UPR + Al_2_O_3_ and UTCFF + UPRCaCO_3_ composites.

**Fig. 3:**
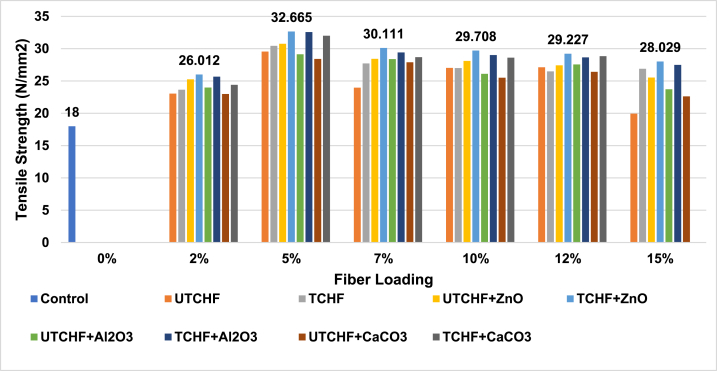
Tensile Strength peaks of Control Sample, UTCHF + UPR, TCHF + UPR, TCHF + UPR + ZnO, TCHF + UPR + Al_2_O_3_ TCHF + UPR + CaCO_3_, UTCHF + UPR + ZnO, UTCHF + UPR + Al_2_O_3_ and UTCHF + UPR + CaCO_3_ composites.

A tensile test was carried out using an all-purpose testing device and the ASTM D 638 standard. All samples were cut into flat forms measuring 150 mm in length, 12 mm in breadth, and 4–4.5 mm in thickness [11]. Tensile Strength is assessed by stress, expressed in force per unit area. Tensile strength calculation formula:

riAny mateal's tensile Strength (TS) is an essential attribute. High tensile strength gives a composite material exceptional fiber strength, superior durability, and improved adhesion between the matrix and dispersion phase. It can be inferred from the analysis of Fig. 2, Fig. 3, and ESI Fig. S_1 and S_2 that the manufactured control sample of unsaturated polyester resin (UPR) in this study had a lower tensile strength value (18 N/mm^2^) than the other animal fiber–based polymer composite samples. All of the figures depict the variance in tensile strength and indicate that 5 % fiber reinforcement produced the most remarkable results.

In comparison to 5 % untreated fiber-based composites (30.23 N/mm^2^ for UTCFF + UPR, 29.58 N/mm^2^ for UTCHF + UPR, 30.99 N/mm^2^ for UT(CFF + CHF) and 22.32 N/mm^2^ for UTLF + UPR), treated fiber-based composites demonstrated improved TS values (32.24 N/mm^2^ for TCFF + UPR, 30.44 N/mm^2^ for TCHF + UPR, 32.97 N/mm^2^ for T(CFF + CHF) and 23.11 N/mm^2^ for TLF + UPR). The treated chicken feather, cow hair, mixed (chicken feather + cow hair), and leather fiber-based composites exhibited higher tensile strength than their untreated counterparts, with improvements of 6.65 %, 2.91 %, 6.39 %, and 3.53 %, respectively. The increased durability is due to the retention of the structural protein, which adds extra firmness to the composites. Even after the fibers undergo chemical treatment and binding agents and impurities are removed, this protein retains its integrity. It can be shown that for up to 5 % of fiber loading, the tensile strength increases and then declines as the fiber content rises. The effective matrix cross-section decreased, and stress concentration rose, which caused reduction in tensile strength when fiber loading was increased. However, chicken feathers significantly increased the strength of polymer composites compared to cow hair and leather fibers. The results showed that cow hair-based composites performed better for the samples created compared to samples reinforced with leather fibers, but composites with chicken feathers and cow hair mixed in showed the highest tensile strength because the UPR's tiny spaces were filled with fibers more densely. The TS value of composites was increased when ZnO, Al_2_O_3_, or CaCO_3_ was employed as filler. Among the composite samples, the combination of treated chicken feather and cow hair fibers (CFF + CHF) with ZnO filler and UPR exhibited the highest tensile strength, reaching 34.95 N/mm^2^. This was a substantial improvement in comparison with the control sample, with a tensile strength of 18 N/mm^2^. Specifically, the increase in tensile strength was 94.17 %. In contrast, the CFF + UPR + ZnO composite showed a 3.04 % improvement, the CHF + UPR + ZnO composite had a 7.04 % improvement, and the LF + UPR + ZnO composite demonstrated a 39.74 % increase in tensile strength when compared to the control.

Due to their distinctive size-dependent optical, electrical, mechanical, chemical and magnetic characteristics, which differ significantly from those of bulk materials, inorganic particles have already established themselves as an essential component for several industrial applications. Because of their effectiveness and broad range of antibacterial properties, inorganic particles, including zinc oxide, aluminum oxide, and calcium carbonate, are very appealing in the composite sector [12]. Over the past few years, ZnO-filled polymer matrix composites have drawn much interest. ZnO nanoparticles can block UV rays. ZnO NPs loaded polymer matrix composites have been reported to have significantly improved mechanical, water vapor barrier, and antibacterial characteristics [13].

When incorporated at a 1 % concentration, ZnO, Al_2_O_3_, or CaCO_3_ significantly enhance the tensile strength of the composite. These inorganic components boosted the tensile strength compared to the base composites. The positive charges of the materials and the negative charges of the chicken feather, cow hair, or leather fiber biopolymer matrix likely electrostatically interacted to increase the tensile strength after the incorporation of the aforementioned inorganic materials, promoting the formation of solid films and more extensive biopolymer helices [14].

### 5.2 Tensile modulus analysis

**Fig. 4:**
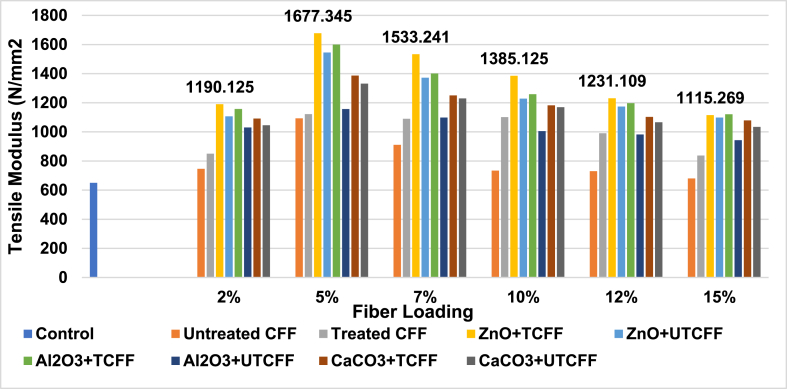
Tensile Modulus peaks of Control Sample, UTCFF + UPR, TCFF + UPR, TCFF + UPR + ZnO, TCFF + UPR + Al_2_O_3_ TCFF + UPR + CaCO_3_, UTCFF + UPR + ZnO, UTCFF + UPR + Al_2_O_3_ and UTCFF + UPR + CaCO_3_ composites.

**Fig. 5:**
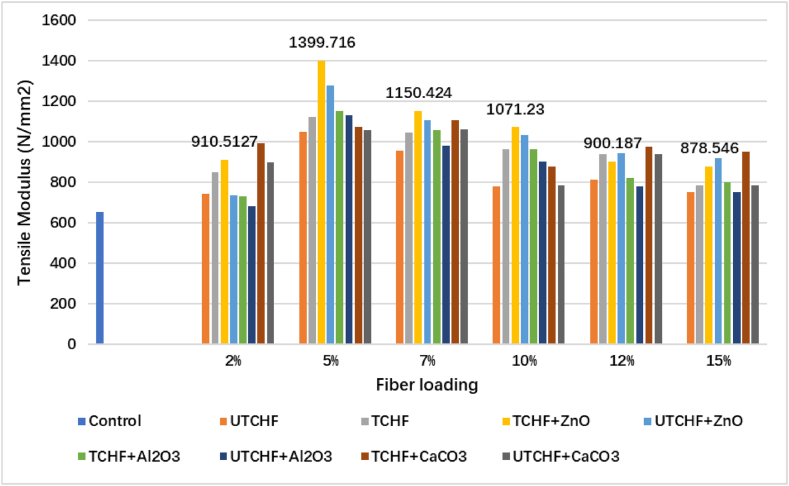
Tensile Modulus peaks of Control Sample, UTCHF + UPR, TCHF + UPR, TCHF + UPR + ZnO, TCHF + UPR + Al_2_O_3_ TCHF + UPR + CaCO_3_, UTCHF + UPR + ZnO, UTCHF + UPR + Al_2_O_3_ and UTCHF + UPR + CaCO_3_ composites.

Fig. 4, Fig. 5 and ESI Fig. S_3 and S_4 depict the variation in the composites' tensile modulus. These results demonstrated that the control sample's TM value is 650 N/mm^2^. The 5 % TCFF and 5 % UTCFF reinforcement produced superior results with values of 1122.01 N/mm^2^ and 1093.14 N/mm^2^ than the control sample, increasing 72.62 % and 68.18 %, respectively, above the control sample. The treated leather fiber, cow hair and chicken feather-based composites had greater tensile moduli than the untreated counterparts, respectively, by 2.64 %, 10.04 %, and 4.16 %. When the fibers underwent chemical treatment, the contaminants and other binding substances were eliminated, leaving only the structural protein, which increased the composites' tensile modulus. Compared to the other animal fiber-UPR reinforced composites, the TCFF reinforcement at 2–15 % fiber loading produced the best results. Chemical treatment, namely 5 % fiber loading, increased the reinforcing of CHF, CFF, and LF-based composites. Fig. 4, Fig. 5 shows the fluctuation in TM caused by chemical treatment and the inclusion of three filler materials: ZnO, Al_2_O_3_, and CaCO_3_. Tensile modulus first increases to 5 % of fiber loaded and then declines as the fiber concentration increases. The decrease in tensile modulus with higher fiber loading was caused by a reduction in effective cross-section of the matrix and an elevation in stress concentration.

The highest observed TM value in the dataset reached 1777.345 N/mm^2^, and this result was explicitly achieved with the combination of treated chicken feather fibers (5 %) and UPR with ZnO filler 1399.716 N/mm^2^ for treated 5 % CHF + UPR with ZnO filler, 1788.741 N/mm^2^ treated 5 % (CFF + CHF) + UPR with ZnO filler and 1512.430 N/mm^2^ for treated 5 % LF + UPR with ZnO filler. These values were 173.43 %, 115.34 %, 175.19 %, and 132.68 % greater than the control sample's value. The highest value found was 1788.741 N/mm^2^ for the treated 5 %(CHF + CFF) + UPR with ZnO filler composite, with improvements of 0.64 %, 20 %, and 11.65 % over TCFF, TCHF, and TLF based composites incorporated ZnO. Among the inorganic materials loaded in UPR, the untreated fiber-added composites with Al_2_O_3_ filler had the lowest value. Chemical treatment enhanced the values of tensile modulus for all fibers-UPR composites. The tensile modulus of composites is significantly affected by a 1 % concentration of Al_2_O_3_, CaCO_3_, or ZnO. Additionally, the mechanical, antibacterial properties and water vapor barrier of ZnO-filled polymer matrix composites exhibit substantial enhancement.

Including fibers in the composite reduced stress transmission between the matrix and the fiber. Conversely, lower and higher fiber percentages might offer insufficient strength to composites. As a result, the TM values of the 2 % and 7%–15 % fiber-based composites were lower.

### 5.3 Elongation at break (%) analysis

**Fig. 6:**
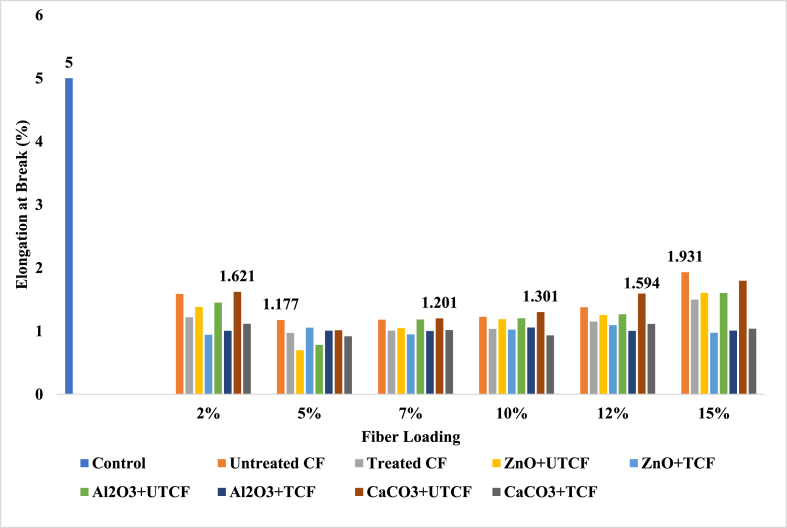
Elongation at Break peaks of Control Sample, UTCFF + UPR, TCFF + UPR, TCFF + UPR + ZnO, TCFF + UPR + Al_2_O_3_ TCFF + UPR + CaCO_3_, UTCFF + UPR + ZnO, UTCFF + UPR + Al_2_O_3_ and UTCFF + UPR + CaCO_3_ composites.

**Fig. 7:**
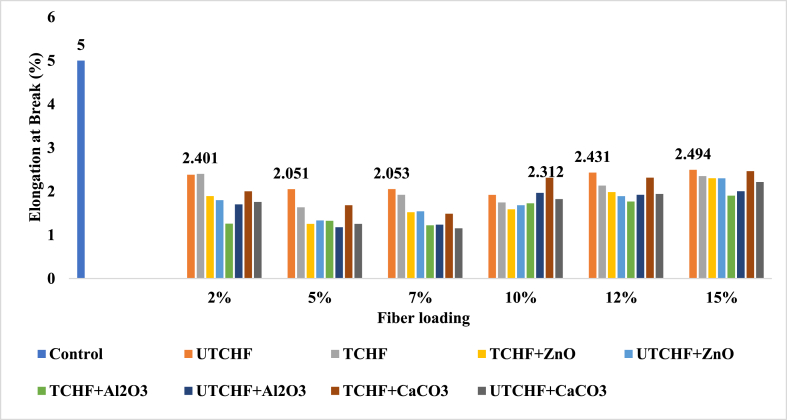
Elongation at Break peaks of Control Sample, UTCHF + UPR, TCHF + UPR, TCHF + UPR + ZnO, TCHF + UPR + Al_2_O_3_ TCHF + UPR + CaCO_3_, UTCHF + UPR + ZnO, UTCHF + UPR + Al_2_O_3_ and UTCHF + UPR + CaCO_3_ composites.

The term “elongation” describes how a material changes in length in response to a tensile tension. A higher % elongation often denotes a higher-grade material when coupled with strong tensile characteristics. Elongation, which evaluates how much bending and shaping a material can tolerate without breaking, is significant in manufacturing. The deformation behavior of the matrix material and the fillers' capacity to absorb energy during deformation both have an impact on the length at break. The elongation at break (Eb) findings for the control sample, CFF-UPR, LF-UPR (CFF + CHF)-UPR, and CHF-UPR composites are visually demonstrated in Fig. 6, Fig. 7 and ESI Fig. S_5 and S_6. Among the test findings, the control sample exhibited the highest EB value at 5 %. In contrast to other composite reinforcements, the UTLF + UPR + ZnO composite, when reinforced with a 2 % fiber loading, yielded the highest results, boasting a value of 2.89 %. This represented a substantial improvement, being 42.20 % lower than the performance of the control sample. Comparing animal fiber-based composites to the control sample UPR, elongation at break was reduced. Chemical processing has frequently been associated with increased elongation at break value. The findings showed that in most cases of elongation at break, the chemical treatment's impact on leather fibers, cow hair, and chicken feathers was less effective. Ductile polymer composites, which have high elongation to break, are made with small, evenly scattered filler particles. As a result of the treatment and the incorporation of filler agents, there was an enhancement in both the TM and TS values, whereas the Eb values decreased. Specifically, in the case of (CFF + CHF) fiber-reinforced UPR-based composites, achieving higher TS and TM values necessitated a trade-off in terms of Eb, indicating that Eb had to be sacrificed to attain the desired TS and TM improvements. When the leather fiber loading was 2 %, the highest elongation at break (2.89 %) was noted. As a result, as the Eb increased, the TS reduced. The highest values of elongation at break were 2.49 %, 1.93 %, and 1.19 % for 15 % CHF, CFF, and (CFF + CHF) loaded composites. From the received data, the average value of elongation at break was better due to the ability of the fillers to absorb energy through deformation and the deformation behavior of the UPR matrix. The CFF and (CFF + CHF) loaded composites gave the average value of elongation; on the contrary, the leather fiber-based composites gave the best mean result because the strength increases, then elongation at break decreases, and vice-versa. Animal fibers were used as a reinforcing filler in this experiment. Therefore, it was anticipated that as tensile modulus and tensile strength increased, the elongation at break values would also decrease. Similar findings have been found in other records [15]. It is evident from this experiment that jute composites significantly outperformed the matrix material (UPR) in terms of strength and modulus values, indicating superior fiber matrix adherence. The toughness, elasticity, and ductility of the composite are all enhanced by an increased elongation at break.

### 5.4 Bending strength analysis

**Fig. 8:**
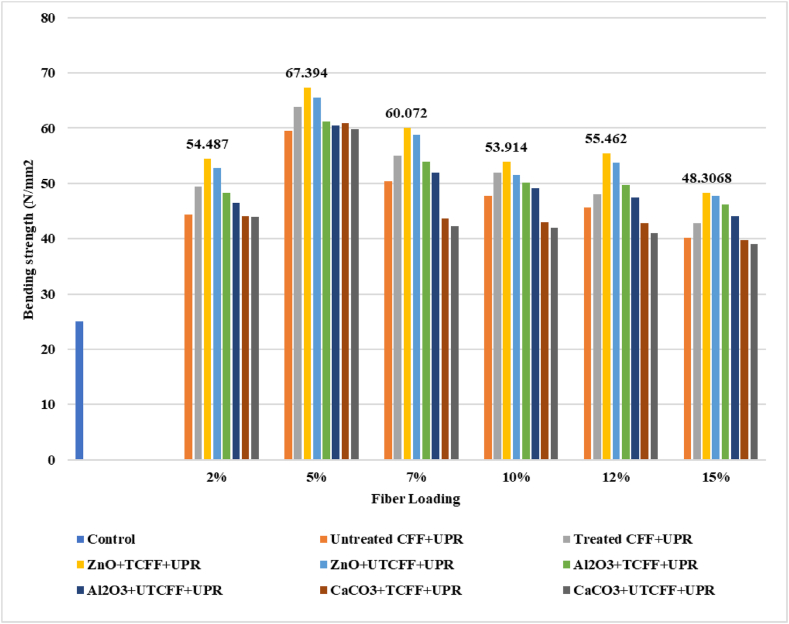
Bending Strength peaks of Control Sample, UTCFF + UPR, TCFF + UPR, TCFF + UPR + ZnO, TCFF + UPR + Al_2_O_3_ TCFF + UPR + CaCO_3_, UTCFF + UPR + ZnO, UTCFF + UPR + Al_2_O_3_ and UTCFF + UPR + CaCO_3_ composites.

**Fig. 9:**
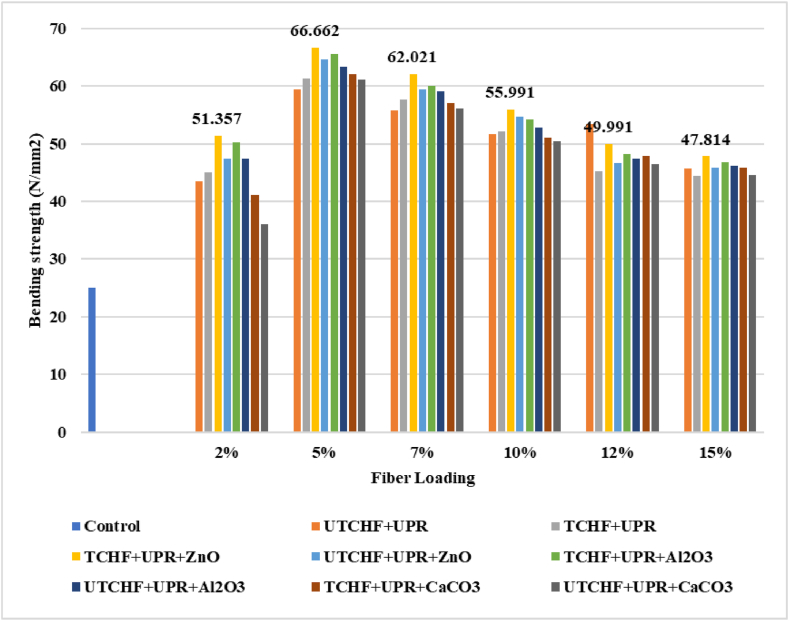
Bending Strength peaks of Control Sample, UTCHF + UPR, TCHF + UPR, TCHF + UPR + ZnO, TCHF + UPR + Al_2_O_3_ TCHF + UPR + CaCO_3_, UTCHF + UPR + ZnO, UTCHF + UPR + Al_2_O_3_ and UTCHF + UPR + CaCO_3_ composites.

The results are presented in the unit of N/mm^2^, as depicted in Fig. 8, Fig. 9 and ESI Fig. S_7 and S_8, illustrating the bending Strength (BS) values for various types of composite samples. Comparing the outcomes of using animal fibers at different percentages, 5 % of each reinforcement gave the greatest results. 5 % of the TCFF and UTCFF loading composites provided BS values of 63.07 N/mm^2^ and 59.52 N/mm^2,^ respectively, which are better by 152.28 % and 138.08 % than the control sample (UPR), which had BS values of 25 N/mm^2^ to present. The chemically fiber-based composites revealed a better result than the untreated fiber-based composites. The improved hydrophilic nature and surface texture of the chemically treated fibers are responsible for the considerable increase in ultimate bending strengths of the produced UPR composites. These characteristics resulted in a high interfacial adhesion between the UPR polymer matrix and the fibers [16]. The BS value of 5 % TCFF, TCHF, TLF reinforced UPR composite incorporated ZnO was observed at 67.39 N/mm^2^, 66.66 N/mm^2,^ and 62.12 N/mm^2^ which is more significant at 169.56 %, 166.64 %, and 148.48 % than the control sample. The highest value of BS was found at 68.91 N/mm^2^ for 5 % treated fiber loading of (CFF + CHF) reinforced with ZnO filling agent composite and increased by 175.62 % to that of the control sample.

When ZnO, Al_2_O_3_, or CaCO_3_ were used as filler, the BS value of composites was improved. Due to their distinctive size-dependent magnetic, optical, electrical, and chemical and mechanical characteristics, which predominantly vary from those of bulk materials, inorganic particles have already established themselves as an essential component for several industrial applications. Because of their effectiveness and broad range of antibacterial properties, inorganic particles, including zinc oxide, aluminum oxide, and calcium carbonate, are very appealing in the composite sector. Over the past few years, ZnO-filled polymer matrix composites have drawn much interest. The repair of resin composite restorations involves the use of an inorganic material agent as well. Applying the filler after surface pretreatments significantly increases the bond strength of the repair restoration. This study demonstrated that chemically treating the reinforcing materials increased their bending Strength. The effectiveness of stress transfer from the matrix to the fiber is highly dependent on the interfacial connection between the two. A strong bond is essential for increasing the TS of the composite. The overall mechanical properties of fiber-reinforced polymeric composites are significantly influenced by the fiber-matrix interface. Both treated and untreated samples exhibited higher values compared to the control samples, indicating a similar trend in the case of tensile strength (TS). The improvements in fiber-based composites may have similar underlying reasons.

### 5.5 Bending modulus analysis

**Fig. 10:**
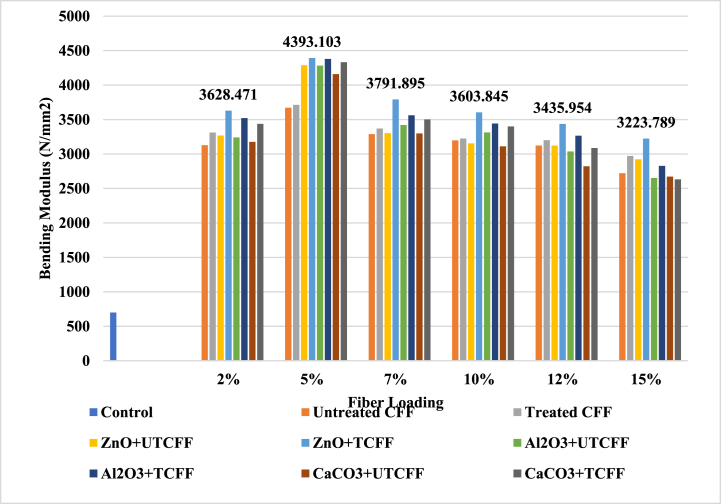
Variation of Bending Modulus peak of Control Sample, UTCFF + UPR, TCFF + UPR, TCFF + UPR + ZnO, TCFF + UPR + Al_2_O_3_ TCFF + UPR + CaCO_3_, UTCFF + UPR + ZnO, UTCFF + UPR + Al_2_O_3_ and UTCFF + UPR + CaCO_3_ composites.

**Fig. 11:**
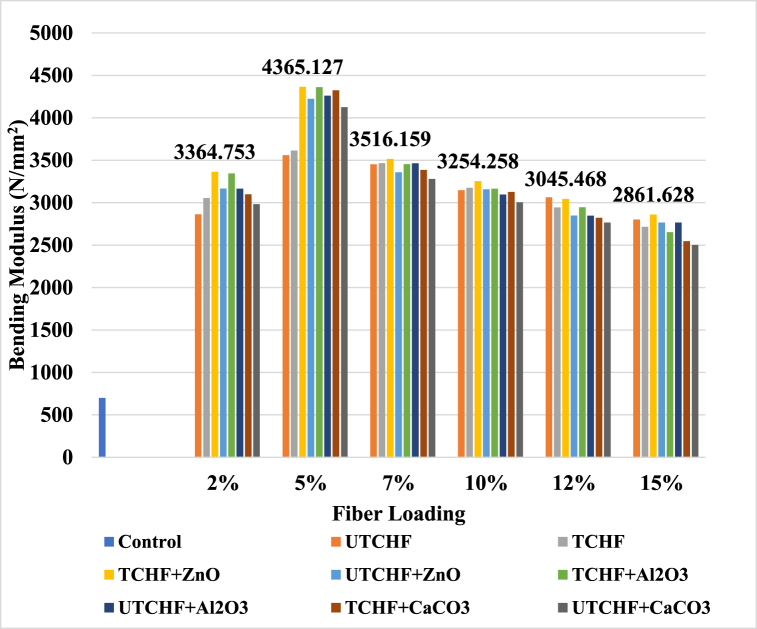
Bending Modulus peaks of Control Sample, UTCHF + UPR, TCHF + UPR, TCHF + UPR + ZnO, TCHF + UPR + Al_2_O_3_ TCHF + UPR + CaCO_3_, UTCHF + UPR + ZnO, UTCHF + UPR + Al_2_O_3_ and UTCHF + UPR + CaCO_3_ composites.

Fig. 10, Fig. 11 and ESI Fig. S_9 and S_10 show a visual representation of bending modulus (BM) for several types of composites. The bending modulus had a similar trend to tensile strength and modulus with varying fiber loading from 0 wt% to 20 wt%. The control sample UPR's bending modulus was 700 N/mm^2^. The maximum values observed in the case of ZnO filler-5% fiber-based treated (CFF + CHF) + UPR composites reached 4400.415 N/mm2, which were approximately 528.63 % larger than the values recorded for the control sample concerning the BM property. Additionally, the composite with a 5 % loading of chemically treated (CFF + CHF) mixed fibers performed 2.53 % better than the composite with untreated fiber loading. The 5 % treated (CFF + CHF) mixed fiber and UPR produced an excellent bonding with each other. Thus, both the constituents of composites can effectively transfer the stress generated due to the applied load. Both bending strength and modulus decrease as the weight percentage of fibers further increases, which is plausibly due to the inadequate bonding between the fiber and the UPR. As usual, the leather fiber-reinforced composites showed the lowest values among the two other employed fibers: cow hair and chicken feather. Based on the comprehensive dataset, composites reinforced with chemically treated chicken feather fibers and unsaturated polyester resin demonstrated the second-highest tensile and bending properties when utilizing a 5 % fiber loading. In contrast, composites incorporating cow hair and leather fibers displayed comparatively lower values in both tensile and bending capabilities.

### 5.6 Effect of gamma radiation on tensile properties of the composite

**Fig. 12:**
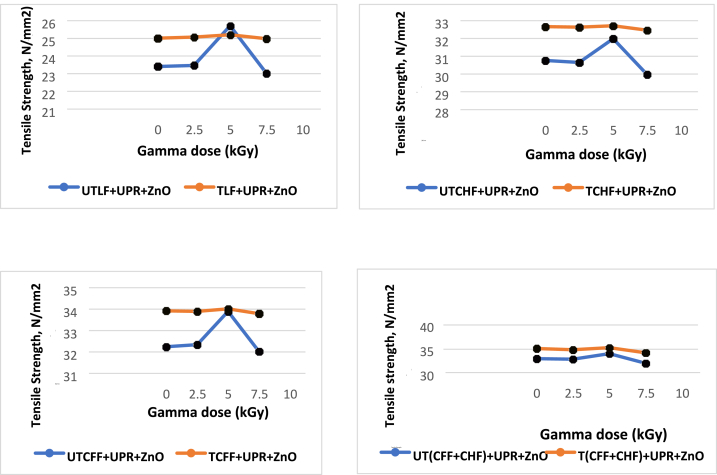
Effect of gamma radiation on tensile strength (MPa) of different animal fiber-based composite.

The tensile Strength (TS) of composites made from animal fiber and UPR increased after being exposed to gamma radiation. In contrast, the TS values of composites without sucrose initially increased but then decreased after reaching a specific radiation dose threshold. The greatest tensile strength value of each utilized 5 % fiber-based composite was disclosed by applying Gamma radiation doses to the treated and untreated fiber-loaded composites. The most favorable outcome was achieved with a 5.0 kGy gamma radiation dose, leading to enhancements of 9.57 % for the UTLF + UPR + ZnO composite, 4 % for the UTCHF + UPR + ZnO composite, 5.11 % for the UTCFF + UPR + ZnO composite, and 3.16 % for the UT(CHF + CFF) + UPR + ZnO composite, respectively. Although the TS for the alkaline treated animal fiber/UPR-based composite fell by a minor amount after a 7.50 kGy dosage, no significant results were identified when the material was exposed to gamma radiation. The chain scission of alkaline-treated animal fiber/UPR-based composites may be to blame—gamma radiation's impact on the hide. Cow hair and chicken feather fiber/UPR-based composite is shown in Fig. 12.

**Fig. 13:**
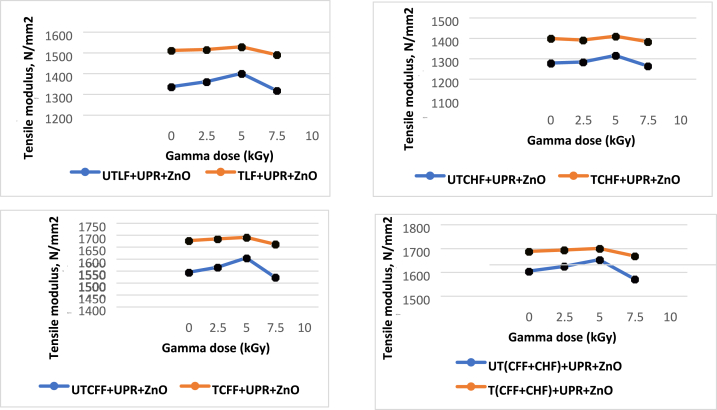
Effect of gamma radiation on tensile modulus of different animal fibers/UPR-based composite.

The TM values rose until a particular dosage of gamma radiation was applied to the composite made from untreated animal fiber/UPR. With a gamma dosage of 5.0 kGy, patients had the greatest improvement at 4.78 %, 2.91 %, 3.89 %, and 2.98 % for UTLF + UPR + ZnO, UTCHF + UPR + ZnO, UTCFF + UPR + ZnO and UT(CHF + CFF) + UPR + ZnO composite respectively. Under gamma radiation, the TM values of the chemically treated animal fibers/UPR-based composite dropped linearly, with the maximum decrease being 1.34 percent of the TM at a dose of 7.50 kGy. One reason could be that the 7.5 kGy gamma dose caused the chemically treated fibers/UPR-based combination to break apart in a longer chain. Fig. 13 shows how gamma rays affect a combination of fibers and UPR.

**Fig. 14:**
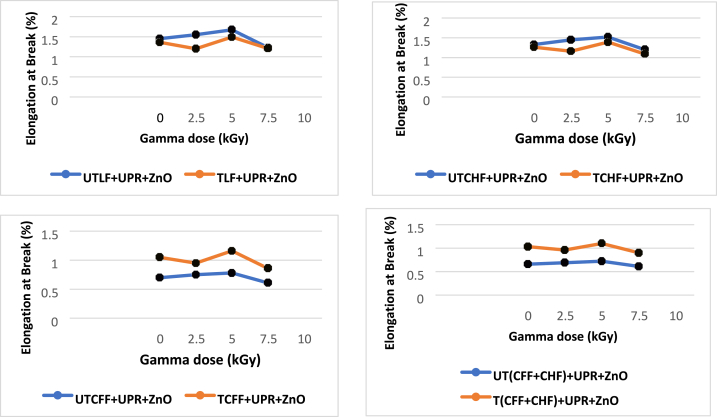
Effect of gamma radiation on elongation at break.

After the application of gamma radiation, the Eb values decreased for the chemically treated animal/UPR-based combination, but they then increased again. The Eb values were initially elevated and subsequently decreased for the materials that were not chemically treated. The fiber-based material not exposed to chemicals responded best to the 5.0 kGy gamma radiation. The improvements were 15.1 %, 14.28 %, 11.43 %, and 9.09 % for the respective composites of UTLF + UPR + ZnO, UTCHF + UPR + ZnO, UTCFF + UPR + ZnO, and UT(CHF + CFF) + UPR + ZnO. The largest reduction in the chemically treated fiber/UPR-based composite was found at 18.10 % of the Eb for TCFF + UPR + ZnO composite at 7.50 kGy dosage. Fig. 14 illustrates how gamma radiation has an impact on the Eb of an animal fiber/UPR-based composite.

Common knowledge suggests that gamma radiation can deposit energy within solid materials through various mechanisms, including the photoelectric effect, Compton scattering, and pair creation. Among these mechanisms, Compton scattering is widely recognized as the primary process responsible for energy transfer when gamma radiation is administered at intermediate energy doses. In the fiber, free radicals are created by gamma radiation. The characteristics of the fiber and composite materials alter due to this newly produced radical. Due to its high-energy nature, gamma radiation does not necessitate the use of a photo-initiator when applied to animal fiber/UPR-based fabrics or composite materials. Comparative data of this study and literature are registered in Table 2.

**Table 2:** Comparative table of the mechanical properties.

Reinforcing material (natural fiber)	Matrix material	Property	Experimental value (N/mm^2^)	References
UTCFF	UPR	TS	30.23	This study
UTCHF	UPR	TS	29.58	
UT(CFF + CHF)	UPR	TS	30.99	
UTLF	UPR	TS	22.32	
TCFF	UPR	TS	32.24	
TCHF	UPR	TS	30.44	
T(CFF + CHF)	UPR	TS	32.97	
TLF	UPR	TS	23.11	
T(CFF + CHF)	UPR + ZnO	TS	34.95	
TCFF	UPR + ZnO	TM	1777.34	
TCHF	UPR + ZnO	TM	1399.71	
T(CFF + CHF)	UPR + ZnO	TM	1788.74	
TLF	UPR + ZnO	TM	1512.43	
TCFF	UPR	BS	63.07	
UTCFF	UPR	BS	59.52	
TCFF	UPR + ZnO	BS	67.39	
TCHF	UPR + ZnO	BS	66.66	
TLF	UPR + ZnO	BS	62.12	
T(CFF + CHF)	UPR + ZnO	BS	68.91	
T(CFF + CHF)	UPR + ZnO	BM	4400.41	
UTCFF	UPR	TS	29.56	[17]
UTCHF	UPR	TS	28.32	[17]
UTLF	UPR	TS	25.94	[18]
TCFF	UPR	TS	27.25	[17]
TCHF	UPR	TS	32.24	[17]
TCFF	UPR + ZnO	TM	1083	[19]
TCHF	UPR + ZnO	TM	1138.54	[18]
TCFF	UPR	BS	60	[19]
UTCFF	UPR	BS	50.89	[20]
TCFF	UPR + ZnO	BS	58	[21]
TCHF	UPR + ZnO	BS	79.88	[18]
Jute	UPR	BS	50	[22]
Banana	UPR	BS	40	[23]
Banana	UPR	TS	40	[7]
Banana	UPR	TM	811	[23]
Coir	UPR	TS	17.92	[24]
Cotton	UPR	TS	112.6	[25]
Corn straw fiber	UPR	TS	8.62	[26]
Corn straw fiber	UPR	BS	50.58	[26]

*TS=Tensile Strength, TM = Tensile Modulus, BS= Bending Strength, BM=Bending Modulus.

### 5.7 Fourier-transform infrared spectroscopy

Identifying the functional groups of the composite materials and unsaturated polyester resin (UPR) was done using Fourier Transform Infrared Spectroscopy (FTIR), and the corresponding values can be found in ESI Figs. S_11-S_14. A prominent peak for the carbonyl (C=O) group was visible on the UPR graph (Fig. S_11) at about 1719 cm^−1^ wave number. Another significant spectrum component is C–*O*–C, which appears at around 1261 cm^−1^. The sp3 bending vibration of the -CH3 groups peaked at around 1450 cm^−1^ region. Additionally, a strong peak at 700 cm^−1^ indicated the existence of = C–H out of plane bending vibration, while a faint peak at 2937 cm^−1^ corresponded to -C-H stretching. Distinct and prominent peaks at 3405 cm^−1^ were observed when polymeric –OH and –NH_2_ groups were present. Furthermore, the presence of carbonyl (C=O) groups was evident in the infrared spectra of chicken feather fiber-reinforced UPR composites, with peaks observed at 1715 cm^−1^ and 1734 cm^−1^. (Fig. ESI S_12). Because of the likely creation of a hydrogen bond between the oxygen atom of the carbonyl group and the hydrogen of the –NH group in keratin, the peaks were slightly displaced from 1719 cm^−1^. In the case of CHF reinforced UPR composites for carbonyl (C=O) groups, the same incident took place for similar H-bonding (Fig. ESI S_13). For fiber-based composites near the wave number 3400 cm^−1,^ broad peaks were found for the presence of polymeric –OH and –NH_2_ groups and little shift from 3405 cm^−1^ due to the cause of formation H-bonding between H of –NH and O of –OH groups. In 1578 cm^−1^ – 1630 cm^−1^ for fibers/UPR composites, medium peaks were found, which is absent in the UPR spectrum. So, it is responsible for the bending vibration of –NH, and this group comes from keratin and collagen. During our FT-IR functional group analysis, we consistently detected the presence of a specific functional group within the 3405 cm^−1^ region. The resulting composite material was expected to contain C–*O*–C and C=C groups originating from the unsaturated polyester resin (UPR). Furthermore, peaks corresponding to C–H, C–O, and –NH, originating from the fibers, were evident in the FT-IR spectrum of the composite sample under investigation. Notably, neither the feather fiber nor the filler materials formed chemical bonds with the UPR or the feather fiber. Instead, the enhanced mechanical properties of the materials were due to the mechanical interaction between the fibers and UPR. These findings align with similar observations reported in published articles.

### 5.8 Scanning electron microscopic (SEM) analysis

ESI Fig. S_15 (a), (b), (c), and (d) show the SEM images of the neat, unsaturated polyester resin matrix, chicken feather fiber, leather fiber, and cow hair in that order. The animal fiber's surface was not entirely smooth, as seen by the SEM picture, which strengthened the mechanical bonding. After being put through a tensile test, the broken samples were chosen for surface morphology study using an SEM machine to examine the matrix adhesion, leather fibers, cow hair, and chicken feathers in the composites. To further understand the interfacial characteristics between the waste fibers of cow hair, leather, and chicken feathers and the matrix, we conducted SEM analysis of the surface topology of the composites. In the SEM image, the inorganic materials (ZnO, Al_2_O_3,_ and CaCO_3_ were dispersed in equilibrium.

The scanning electron microscope (SEM) was used to examine the interface between the phases of leather fiber, cow hair fiber, and chicken feather fiber and the unsaturated polyester resin (UPR) matrix on the fracture surface of the manufactured composite samples. The visual representation of the resulting composite's morphological structure was presented in ESI Fig. S_15 (a-k). These images illustrated a solid and intimate bond between the matrix and the fibers. Notably, up to a specific fiber-loading threshold, the SEM analysis revealed a reduced occurrence of fiber pullout from the matrix on the fracture surface. This phenomenon contributed to the betterment of the composites' mechanical properties.

**Fig. 15:**
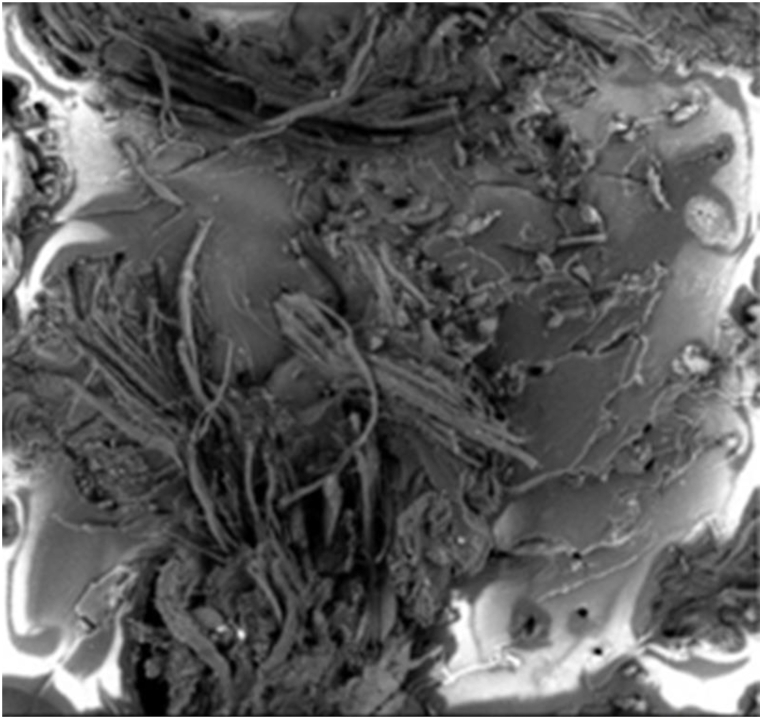
SEM image of TLF + CaCO_3_+UPR.

The SEM pictures also revealed the poor pulling of the fibers from composites. The SEM images of composite materials clearly demonstrated the strong connection between cow hair and UPR. This was evident when the tensile strength test caused a few cow hair fibers to be pulled away from the UPR. Therefore, the physio-mechanical properties can be improved by combining waste materials from the poultry, tannery, and cow hair industries with unsaturated polyester resin. There was no vacant region to be seen in the created composites. The blank spaces shown in the images [ESI Fig. S_15 f, k)] were used to illustrate the fiber withdrawal from the matrix. SEM images of composites showed interrupted bonding. Fig. 15 is the SEM image of TLF + CaCO_3_+UPR.

Due to the close integration of fiber and matrix, it is evident that the mechanical properties of composites surpass those of the control sample. Similar levels of adhesion between the matrix and fiber have been observed in natural fiber-reinforced composites and have been documented previously. Cracks are rarely observed in animal fiber-reinforced composites. However, some cracks were detected in the SEM images after conducting tensile testing. This research primarily focused on physical and mechanical qualities and environmental problems. The characteristics were enhanced by the fiber addition up to a specific proportion. The transmission of force from the matrix to the reinforcing agent was increased through the gradual introduction of fibers to the unsaturated polyester resin matrix. This enhanced force transmission between the fiber and matrix led to a discernible improvement in the mechanical characteristics. The inclusion of filler helped to improve the fiber-matrix interaction, and the improved bonding increased the mechanical characteristics up to a point. In each example, some voids were left by pulled-out fibers, but the fibers all remained lodged in the resin. The lack of a new bond in the FT-IR spectrum suggested that there was no chemical interaction between the fibers and UPR. SEM analysis of the treated chicken feather fiber reinforced UPR composites revealed that at five weight percent of fiber reinforcement, the fibers can effectively transfer stress between themselves and the UPR matrix, resulting in increased tensile strength.

Additionally, a fiber misalignment and entanglement were visible. In terms of the general characteristics of composites, fiber alignment considerations are pretty important. This fiber entanglement may result in resin-rich regions, which may aid in developing pores and voids. Similar SEM picture types have been described in various publications [[27], [28], [29]].

## 6 Conclusion

Composites were fabricated successfully using chicken feathers, cow hair, and leather scraps as reinforcing materials in a synthetic polymer matrix, unsaturated polyester resin. Up to 5 % natural fiber loading in the matrix, the mechanical properties such as bending modulus, bending strength, tensile modulus and tensile strength were increased for all the fabricated composites. The application of 5.0 kGy of gamma radiation dose resulted in improved mechanical properties of the mentioned composites. No significant changes were observed in the FTIR analysis, indicating that no chemical bonds were formed or broken. SEM images confirmed strong bonding between the reinforcing materials and the matrix. To achieve slightly higher mechanical properties, it is recommended to use 5 % natural fiber in the synthetic polymer matrix and apply a 5.0 kGy gamma radiation dose during composite fabrication. This research will minimize the use of synthetic polymer, and environmental pollution will also be decreased by using natural fiber waste. The addition of biodegradable polymers will definitely help in achieving a sustainable environment. A certain percentage utilization of natural fiber in composite fabrication will curtail the amount of synthetic polymer in nature.

## Data availability statement

Data will be made available on request.

## CRediT authorship contribution statement

**Md. Farhad Ali:** Writing – review & editing, Writing – original draft, Methodology, Investigation, Funding acquisition, Formal analysis, Data curation. **Md. Sahadat Hossain:** Methodology, Investigation, Conceptualization. **Israt Jahan Lithi:** Writing – review & editing, Visualization. **Samina Ahmed:** Validation, Supervision, Resources, Project administration, Funding acquisition. **A.M. Sarwaruddin Chowdhury:** Validation, Supervision, Project administration.

## Declaration of competing interest

The authors declare that they have no known competing financial interests or personal relationships that could have appeared to influence the work reported in this paper.
